# Silver Nanocoating Technology in the Prevention of Prosthetic Joint Infection

**DOI:** 10.3390/ma9050337

**Published:** 2016-05-05

**Authors:** Jiri Gallo, Ales Panacek, Robert Prucek, Eva Kriegova, Sarka Hradilova, Martin Hobza, Martin Holinka

**Affiliations:** 1 Department of Orthopaedics, Faculty of Medicine and Dentistry, Palacký University Olomouc, I. P. Pavlova 6, Olomouc 779 00, Czech Republic; martinhobza@gmail.com (M.H.); mholinka@seznam.cz (M.H.); 2 Regional Centre of Advanced Technologies and Materials, Palacký University Olomouc, Šlechtitelů 27, Olomouc 783 71, Czech Republic; ales.panacek@upol.cz (A.P.); robert.prucek@upol.cz (R.P.); sarka.hradilova@upol.cz (S.H.); 3 Department of Immunology, Faculty of Medicine and Dentistry, Palacký University Olomouc, Hněvotínská 3, Olomouc 779 00, Czech Republic; eva.kriegova@email.cz

**Keywords:** prosthetic joint infection, biomaterial-associated infection, anti-adhesive, anti-biofilm, antibacterial surface treatment, silver nanocoating, silver nanoparticles

## Abstract

Prosthetic joint infection (PJI) is a feared complication of total joint arthroplasty associated with increased morbidity and mortality. There is a growing body of evidence that bacterial colonization and biofilm formation are critical pathogenic events in PJI. Thus, the choice of biomaterials for implanted prostheses and their surface modifications may significantly influence the development of PJI. Currently, silver nanoparticle (AgNP) technology is receiving much interest in the field of orthopaedics for its antimicrobial properties and a strong anti-biofilm potential. The great advantage of AgNP surface modification is a minimal release of active substances into the surrounding tissue and a long period of effectiveness. As a result, a controlled release of AgNPs could ensure antibacterial protection throughout the life of the implant. Moreover, the antibacterial effect of AgNPs may be strengthened in combination with conventional antibiotics and other antimicrobial agents. Here, our main attention is devoted to general guidelines for the design of antibacterial biomaterials protected by AgNPs, its benefits, side effects and future perspectives in PJI prevention.

## 1. Introduction

Prosthetic joint infection (PJI) is a feared complication of modern orthopaedic surgery that substantially increases morbidity and even mortality following total joint arthroplasty (TJA) [1,2]. Generally, PJI leads to implant removal and long-term antibiotic therapy with a permanent, increased risk for PJI development in affected patients [3].

Current estimates suggest that up to 3% of primary hip and knee arthroplasties [4], up to 15.4% of revision hip and 25% of knee arthroplasties are complicated by PJI respectively [5]. According to some authors, these numbers are not only underestimated but they are also on the rise [6]. The annual cost of infected revisions in hospitals of the United States of America (USA) could increase from $566 million in 2009 to $1.62 billion by 2020 [7]. As a result, therapy of PJI continues to be associated with enormous costs.

The first postoperative months are the most typical period of PJI manifestation [8] with the incidence rate of late PJI in hip and knee arthroplasty at about 0.07% per prosthesis-year and a higher risk in knee arthroplasties when compared to hip [9].

The leading causes of PJIs are *S. aureus* and coagulase-negative staphylocci followed by streptococci and enterococci (all of these account for approximately 10% of PJI cases) [10,11]. Importantly, the prevalence of methicillin-resistant *S. aureus* (MRSA) in PJI is increasing, especially in the USA [12]. In addition, polymicrobial infections can occur in up to 15% of cases [13] despite the fact that some authors reported a substantial increase in the yearly occurrence of polymicrobial infections over the period of six years (2004 to 2010) with a greater increase in the proportion of gram-negative bacteria during the same period [14].

## 2. Pathogenesis of PJI

The distribution of PJI in time strongly points to the causative link towards surgery and the early postoperative period. A basic prerequisite for PJI development is the size of the bacterial load influencing the operating wound, immune response and the implant. The last two decades were under strong dominance of Gristina’s concept of “race for the surface” [15]. Accordingly, host and bacterial cells compete in determining the ultimate fate of the implant, when host cells colonize the implant surface first, the probability of attachment of bacterial cells is very low and vice versa. However, Gristina’s model is not able to predict PJI in “less clear” situations when the host cell coverage of an implant surface is incomplete and thus offering some places for bacteria adhesion. In addition, some prosthetic surfaces, either articulating or non-articulating, preclude host cell adhesion and development of a protective host film. This model can also be criticized for static conditions because fluid waves occurring many times per hour are typical for TJA. Finally, immune and host tissue responses contribute to the protection of an implant surface to a greater extent than only in terms of simple mechanistic competition for an implant surface. Despite the fact that not all the critical pathogen and host steps/factors have been elucidated to date [16], for instance an infection dose no doubt plays an important role. A higher bacterial load of *S. aureus* could alter the host immune response and accelerate biofilm formation [17] while a low level of “appropriate” bacterial contamination might even serve as a potent immunomodulatory factor preventing the development of PJI (“implant infection paradox”) [18]. Some evidence also suggests the role of genetic susceptibility [19,20]. Taken together, instead of Gristina’s metaphor, a specific local immunologic and tissue constellation type of pathogen as well as bacterial load interplay with each other and influence the implant-tissue interactions, either towards non-infective or infective statuses.

**Figure 1:**
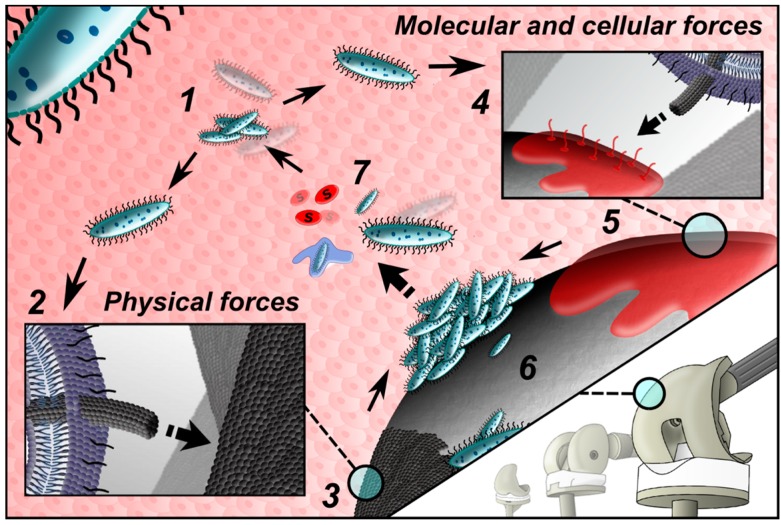
Free-floating bacteria (**1**) come close to the implant surface, here they interact via a set of chemical and physical mechanisms with a biomaterial surface covered by host cells/proteins. The majority of bacterial pathogens express specific surface adhesion molecules called adhesins (bacteria may have multiple adhesins for different surfaces); bacterial adhesion can be described as having an initial reversible, predominantly physically driven phase (**2**) and a time-dependent and irreversible molecular and cellular phase (**4**). The former is realized by Brownian motion, van der Waals attraction forces, gravitational forces, surface electrostatic charge and hydrophobic interactions (**3**); the latter employs a selective bridging function of bacterial surface polymeric structures, which include capsules, fimbriae or pili and slime; intermolecular interactions are facilitated by a protein film covering an implant immediately after its placement into the host body (**5**). Firm sticking of bacteria to the biomaterial surface allows them to create colonies (**6**) with biofilm formation, which is associated with a continuous release of free floating bacteria and signaling molecules (**7**).

The most destabilizing factor is the basic yet highly successful survival strategy of bacteria in general: their ability to adhere and survive on virtually all natural and synthetic surfaces (Figure 1) [21,22]. The bacterial cell membrane contains various types of adhesins for a wide range of biomaterial surface receptor sites, members of the family of Microbial Surface Components Recognizing Adhesive Matrix Molecules (MSCRAMMs) [23]. Environmental and surface characteristics of a biomaterial such as surface roughness, hydrophobicity and electrostatic charge play only conditional roles [24]. A reservoir of receptors for bacterial adhesive ligands mediating adhesion of free-floating bacteria to the surface of the biomaterial, offers a conditional protein film covering the implant immediately after its placement into the host body [25]. The spectrum of binding molecules depends at least partly on the particular type of biomaterials attracting an exact set of host proteins and lipids [26,27,28].

Conceptually, the process of bacterial adhesion can be divided into two basic phases: reversible, and irreversible [29,30]. The former is mechanically and biologically less stable than the latter. The explanation lies partly in the origin of nonspecific interactions between the implant surface characteristics and bacterial surface adhesins, followed by molecular and cellular interactions closely associated with expression of biofilm specific gene clusters in reversibly attached bacteria [31]. At least four distinct classes of surface proteins have been identified to participate on firm adhesion of *S. aureus* micro-colonies to a biomaterial and to each other [32]. The adhesion phase is followed by gene expression for secretion of protective slime. This process makes bacteria extremely resistant to both the host immune system and antibiotic diffusion [30,33]. The transition between the reversible and irreversible phases of biofilm formation coupled with a phenotypical change, is the last window of opportunity for clinically reasonable preventative measures. Other parameters of biofilm formation are described in detail elsewhere [34], as well as the ability of bacteria to combine different pathogenic strategies [35].

In the host site, the details of tissue integration of a biomaterial are still poorly understood [36,37]. It is believed that immune as well as tissue resident cells recognize an implant surface and orchestrate the processes, leading to periprosthetic bone/soft-tissue regeneration and remodeling, preventing the development of biofilm in the majority of patients [38,39]. However, neither osseointegration nor fibrous tissue encapsulation of large non-fixation parts of an implant can eliminate long-term survivorship of bacterial micro-colonies. Moreover, the peri-implant fibrous barrier impedes contact between the host immunity sentinel cells and bacterial molecules. This interaction is critical for host immune responses dependent on recognition of bacterial pattern-recognition receptors (PRRs; also microbe associated molecular patterns = MAMPs). Importantly, it has been demonstrated that implantation of a medical device impairs the innate local host response and may facilitate the development of PJI [40,41].

As the majority of operating rooms are contaminated within the first few hours of service [42,43], most surgeries are not performed in a bacterial-free environment. All patients are exposed to the same environment within a particular operating room. The question therefore arises as to why some patients go on to have infections and others do not. There is a growing body of evidence that PJI results from a relatively unclear and perhaps unique combination of environmental and genetic factors. The environmental ones could be linked to immune and non-immune factors affecting host response to bacterial load (age, gender, malnutrition, weight, diabetes mellitus, smoking *etc.*); the factors related specifically to implant facilitating for instance, adhesion of bacteria and those related to the surgeon and surgery (operating skills, operating room parameters, surgical time *etc.*). The host genetics strongly influences an individual’s susceptibility to infectious diseases and there is some evidence available for genetic susceptibility to PJI [19].

As a result, there is a strong need for intrinsic implant surface antibacterial functionality that can protect the implant surface from a perioperative attack of pathogenic bacteria as well as help to overcome implant-induced defects in the local immune response.

## 3. Rationale and Basic Concepts of PJI Prevention

Strategies relying on a decreased bacterial load and creating a bacteria-free environment around an implant during the perioperative period are widely implemented in clinical practice [44,45]. There is sufficient evidence supporting systemic [46] and in some cases local ***antibiotic prophylaxis*** [47]. However, the optimal protocol for individual clinical situations is not known yet. At present, antibiotics are administered to all the patients undergoing TJA regardless of the individual risk for PJI development, at least in terms of the beginning, the type of antibiotic and the duration of antibiotic prophylaxis. With regard to the latter, the 24 h regime does not cover the time needed for early wound stabilization, or the period of time the suction drain is in contact with joint and deep tissues. In addition, the increasing occurrence of antibiotic resistance has been recognized to be a global problem. There is also some evidence for selecting antibiotic-resistant staphylococci in relation to wide-range antibiotic prophylaxis [48].

Attempts at formulating evidence-based standards for good clinical and logistic practice in orthopaedic ***operating rooms*** have been made [45,49]. There is a growing pressure on surgeons to improve their surgical skills in order to minimize the surgery-related factors. Educational programs aimed at educating/training orthopaedic surgeons (and all staff) in perioperative strategies of PJI prevention are under way [50].

Finally, strategies based on ***identification of risk patients and optimization of their conditions*** to decrease the probability of PJI development have been proposed. Even though modifiable PJI risk factors have been identified and well-described [50,51], it is often not possible to avoid operating “risk” patients who are not “optimized”. For instance, significant obesity precluded the indication for total hip or knee arthroplasty in some countries several years ago. However, it is unethical to reject surgery in these patients today, despite the fact that they have an increased risk for PJI [52]. Research testing is assessing whether the risk for PJI could be decreased after preoperative immunization of the patients at-risk, by a vaccine that targets either the most frequent pathogen as *S. aureus*, or the key molecules of bacterial adhesion and biofilm formation [53]. 

**Figure 2:**
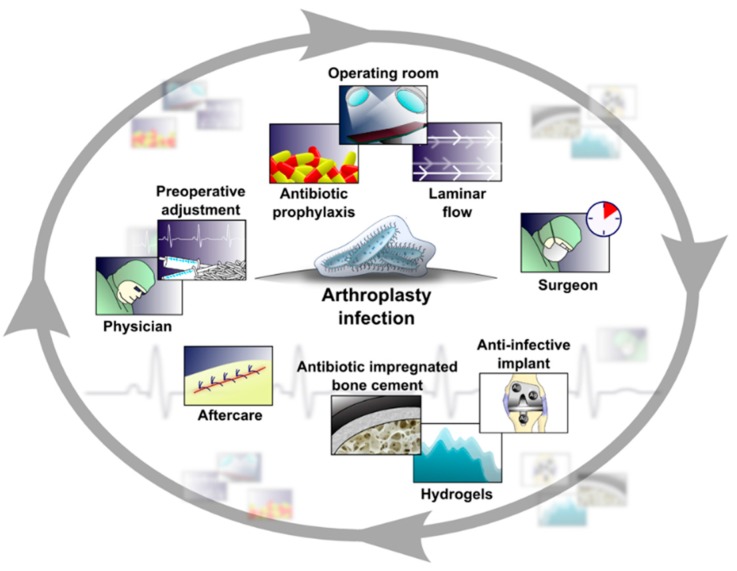
Prevention of PJI consists of a list of measurements optimizing host status/preparedness for surgery (identification of host risk factors, determination of host comorbidities; local antibacterial activities); reducing bacterial load during the surgery (intravenous antibiotic prophylaxis, operating room environment/traffic/management, surgical experience, measurements/tools preventing deliberation of bacteria from the surgeon/operating room personnel, protection of the implant from bacterial contamination/adhesion) and minimizing the chance for postoperative bacterial contamination (wound care strategy, rapid optimization of postoperative immune and metabolic conditions, early ambulance, experienced physiotherapy, eradication of local infections and haematogenous sources of bacteria).

Taking into account the weaknesses associated with all the current preventative strategies, leaders in the field recommend a multistep preventative concept (Figure 2) covering simultaneously all the well-known targets, including the “anti-infective implant” [54,55,56].

## 4. Indications for Implants with Antibacterial Surface Treatment

In accordance with the evidence-based medical rules, it would be relevant to calculate the number of PJIs prevented, by usage of implants with an antibacterial surface. Theoretically, all the patients undergoing TJA are at risk for PJI. Revision cases carry an increased risk, partly due to the prolonged operating time during revision surgeries, in conjunction with a suboptimal local tissue environment [57]. Moreover, there is some evidence that the risk of PJI across the board in orthopaedic surgery, is on the rise [6]. As a result, one could argue that all patients should benefit from implants coated with a proven anti-infective surface. On the other hand, the risk for PJI is not homogenously distributed among arthroplasty patients [50]. Therefore, it might be convincing to implant “biofilm resistant” prostheses only in patients at an increased risk of PJI [51,58]. However, a validated tool for screening patients for an increased risk of PJI does not currently exist. Taken together, the preventative strategy involving all the patients undergoing primary and revision TJA seems to be more justifiable than a more restrictive approach targeting the high-risk patients. However, prior to implementation of such devices, it is necessary to demonstrate the significant reduction of PJI in a well-done, population-based, cost-benefit analysis [38]. An important consideration in designing implants with antibacterial coating relates to the characterization of reasonable and justifiable costs.

## 5. Recommendations for Construction of Implants with Anti-Infective Surfaces

A wide spectrum of substances and technological approaches has been proposed and tested for antibacterial features in orthopaedics (Table 1). In order to fully discuss and evaluate surface treatment technologies it is essential to review the strict criteria related generally to the process of innovation in this field. The requested parameters are as follows:

1. ***biocompatibility*** (the ability of a material to work efficiently with an appropriate host response in specific applications) [59];
2. ***strong evidence of anti-infective efficiency*** (the anti-bacterial efficiency should be demonstrated *in vitro*, *in vivo* and also in an appropriate model of PJI) [60,61,62];
3. ***fixation properties cannot be compromised*** (the antibacterial coating must not compromise long-term stable implant osseointegration or cement fixation);
4. ***durability of the anti-infective effect*** (while clear recommendations are lacking the epidemiological viewpoint suggests that at least two years would be appreciated) [63,64];
5. ***mechanical characteristics of the antibacterial coating*** (resistance to mechanical stresses and strains either during surgery or postoperatively) [65].

Recently, a new classification of the implant-related antibacterial strategies has been proposed distinguishing:

1. ***passive surface finishing/modification*** (PSM);
2. ***active surface finishing/modification*** (ASM);
3. ***perioperative antibacterial local carriers or coatings*** (LCCs) [56].

If the active substance is released from the surface of the implant, over time it may lead to its exhaustion and thereby, a loss of efficiency. It is therefore extremely relevant to design surface modifications with minimal but effective release of active substances into the surrounding tissue, thereby achieving a long, or even indefinite period of effectiveness. This approach may ensure antibacterial protection throughout the life of the implant. A specific set of problems are related to fluid dynamics and adhesion of host proteins, lipids, cells to “active” implant surfaces, limiting their antibacterial efficacy.

## 6. General Remarks on Prosthetic Implant Surface Modifications

**Figure 3:**
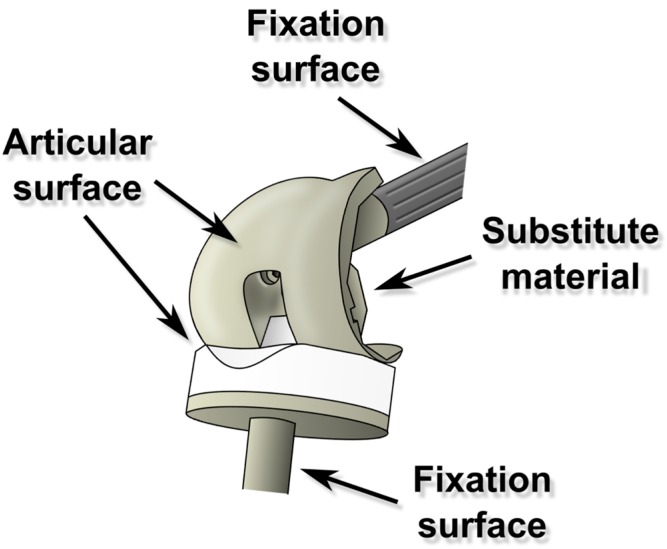
Total joint arthroplasty has several types of surfaces according to their locations and functions; ideally the whole implant should be covered via application of an antibacterial strategy; however, in practice the antibacterial strategy for a particular kind of surface has to respect its critical characteristics (for example the strategy for an articulating surface, let’s say a polyethylene one, has to be different from a non-articulating metallic one).

Polyethylene, a modern generation of zirconia treated ceramic, stainless steel, cobalt-chrome and titanium alloys are the most commonly used materials in TJA implants. In TJA, each material/surface modification has its specific role (e.g., an articulating or a fixation surface) and can occupy a different place in the bulk implant (Figure 3). These parameters together, define the requirements for particular surface modifications in specific implant sites.

A number of principles from basic research have been proposed for translation into technologies potentially suitable for antibacterial treatment of orthopaedic implants [149]. It is easy to distinguish between technologies offering ***anti-adhesive properties***, those working as ***antimicrobial agents*** and those ***combining the above-mentioned approaches***. Anti-infective surfaces can be classified as **“*contact killing” and antimicrobial agent eluting*** respectively [150].

Antibacterial surface technologies can employ ***metals*** (silver, zinc, copper, zirconium *etc.*), ***non-metal elements*** (e.g., selenium), ***organic substances*** (antibiotics, anti-infective peptides, chitosan, other substances) and ***their combinations***. Antibacterial activity of the majority of metal coatings is closely linked to the ionic or nano-form, rather than to the bulk material [151]. ***Nanostructured surfaces and coatings*** (either of inorganic or organic origin) are therefore of great interest. Consequently, the nanoscale surface patterning methods have been applied to fabricate different nanopatterns (e.g., ordered stripes, pits, pillars or squares). Several studies have demonstrated that nanopatterning in conjunction with other surface treatment can inhibit bacterial adhesion [152,153].

**Figure 4:**
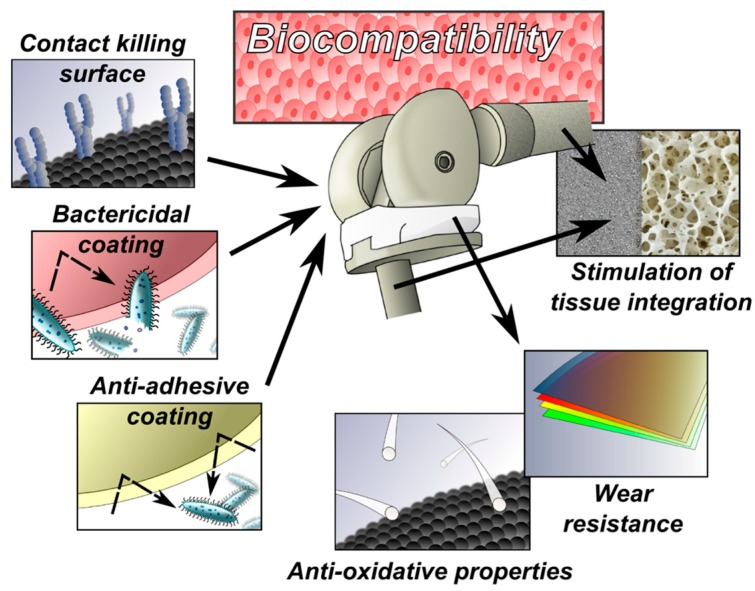
A particular implant surface has to address several implant-related tasks simultaneously and continuously (in the ideal case), therefore engineers have to solve the problem of how to bind (attract, fix) often contradictory functionalities via specific modifications/treatments in the particular surface location.

In terms of functionality, one may divide surfaces as ***mono-functional and multi-functional***. The latter are expected to target multiple biological tasks simultaneously (Figure 4), orchestrating early/long-term tissue adaptation to an implant, facilitating osseointegration and regulating the anti-infective immune response, all in addition to the “intrinsic” antibacterial surface effect [154]. ***Smart surface*** could be a completely different methodology designed to be a self-responsive multitask micro-machine that releases antimicrobial (and other) substances, after stimulation by microbial (or other) signals [155].

## 7. Why Silver Nanoparticle Technologies on the Implant Surfaces?

Currently, AgNP technology is receiving much interest in its use on implant surfaces, mainly for its antimicrobial properties and strong anti-biofilm potential together with relatively low cytotoxicity to mammalian cells. ***AgNPs effectively inhibit the growth of bacteria*** including highly resistant strains at very low concentrations in units of mg/L [92,156,157,158,159,160,161,162], whereas such concentrations do not exhibit an acute cytotoxic effect, which was proved at the concentrations higher than 20 mg/L [163,164,165].

Moreover, in the case of AgNPs, bacterial resistance has not been reported up to now, despite the fact that resistance to ionic silver has been observed. The multilevel antimicrobial (broad target attack) mode of AgNPs ensures that resistance cannot be easily acquired by single point mutations in contrast to antibiotics. Having ***a very low risk of development of bacterial resistance*** it is therefore relevant to know the antibacterial effects of AgNPs. This is an extremely valuable effect especially today, when we are facing growing antibacterial resistance observed in antibiotics and other antibacterial substances. Some experts even refer to the current state as to a “worldwide calamity” or “antibiotic resistance crisis”. Therefore, a joint multilevel and global interdisciplinary action including substituting antibiotics by non-antibiotic approaches could decrease the range and rate of bacterial resistance. 

Moreover, AgNPs have a ***strong anti-biofilm potential*** [162,166,167,168,169,170,171,172,173,174,175,176,177,178,179]. Therefore, these are potentially very attractive for surface protection of orthopaedic implants since PJI is biofilm driven in the majority of clinical cases. As a result, silver is the most prevalent metal used in biomedical applications for antibacterial coating of prosthetic metal implants [180,181,182,183,184,185,186,187,188,189,190]. Both uncoated and coated AgNPs on various surfaces, such as titanium surfaces or catheter surfaces, thoroughly inhibit both planktonic and biofilm-forming bacteria [94,167,191,192]. Saleh *et al.* reported that biofilm and planktonic *E. coli* and *P. aeruginosa* cells showed very similar tolerance to AgNPs upon exposure [191]. Agarwala *et al.* reported high antimicrobial activity on catheters loaded with AgNPs towards planktonic as well as biofilm-forming cells [167]. Similarly, Zhong *et al.* and Harraser *et al.* reported that AgNP-loaded titanium can kill planktonic and adherent bacteria during 1, 4 and 12 days with similar effectiveness [94,192]. On the other side, several studies showed that biofilms decreased susceptibility to AgNPs compared to planktonic cells [193,194]. Choi *et al.* found that biofilms were four times less susceptible to AgNP exposure than planktonic cells were [194]. Starch-coated NPs reduced *P. aeruginosa* and *S. aureus* biofilm growth but completely inactivated planktonic cells at the same AgNP concentrations [195]. It is known that both planktonic and biofilm-forming bacteria produce extracellular polymeric substances (EPS), which has been proved to lower the diffusion rate of NPs [196]. EPS production is much greater in biofilms compared to planktonic bacteria and therefore may provide some protection to biofilm-forming cells from NPs.

## 8. Synthesis of Silver Nanoparticles on the Implant Surface

There are several approaches to synthesize the AgNPs on the implant surface. In the study [87], TiO_2_ (titanium dioxide) nanotubes (NT) on a titanium (Ti) surface were prepared by anodization of the Ti surface and consequently AgNTs were generated on the NT surface by ultraviolet reduction of silver ions. The TiO_2_-NTs loaded with Ag (silver) exhibited a strong antibacterial activity against methicillin-resistant *S. aureus* (MRSA, ATCC43300) *in vitro* for 30 days.

The cathodic arc silver plasma immersion ion implantation process can serve as another method of preparation and immobilization of AgNPs on a Ti surface. The immobilized AgNPs offered good defense against multiple cycles of bacteria (*S. epidermidis*) attacks *in vitro* and the mechanism was independent of silver release [197].

Also Pulse DC magnetron sputtering can be utilized for a generation of AgNPs on a Ti surface, where nanostructured Ti-Ag coatings with different Ag contents (1.2% to 21.6%) are able to kill *S. aureus* effectively during the first few days and remain moderately antibacterial after immersion for 75 days. Compared to pure Ti, the Ti-Ag coatings show good cytocompatibility as indicated by good osteoblast adhesion, proliferation, intracellular total protein synthesis and alkaline phosphatase activity [198].

AgNPs with the size of 50 nm can also be incorporated into a dopamine-modified alginate/chitosan (DAL/CHI) polyelectrolyte multilayer to modify titanium alloy surfaces. The polyelectrolyte multilayer coating enhanced wet ability of titanium alloy and promoted the fibroblast proliferation significantly, which could be attributed to the excellent biocompatibility of DAL/CHI [199]. Despite the slight fall of L929 cell activity after AgNP incorporation, AgNP-DAL/CHI multilayer inhibited the growth of both *E. coli* and *S. aureus* [199].

Hexagonal closed-packed TiO_2_ nanotubes with the diameter of 30–100 nm were prepared by anodization of a Ti foil, where the size of nanotubes was dependent on the parameters of anodization [200]. The size and shape of the generated AgNPs (12–40 nm) on TiO_2_ nanotubes by UV (ultraviolet) irradiation depends mainly on the size of TiO_2_ nanotubes and silver ion concentration. The highest antibacterial activity was obtained for TiO_2_ nanotubes with the opening diameter of about 100 nm and AgNPs with an average size of 20 nm, whereas good cell viability using osteoblast MG63 cells was remained [200]. The Ti/TiO_2_ nanotubes/AgNPs composites can also be prepared with the assistance of quaternary ammonium salt (QAS, 3-trimethoxysily-propyldimethyloctadecyl-ammonium chloride). The Ag nanoparticle loaded and QAS coated TiO_2_ nanotube substrates demonstrated long-term antibacterial effect and displayed good biocompatibility [201].

## 9. Antibacterial Effect of Silver Nanoparticles

**Figure 5:**
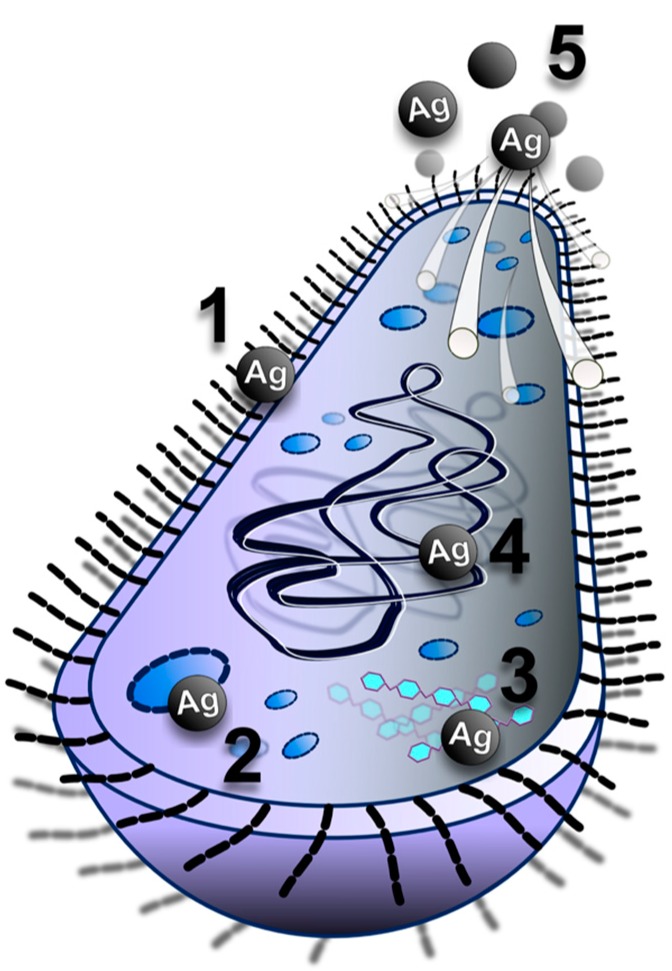
Silver nanoparticles simultaneously target bacteria via the destruction of their wall, inactivation of their enzymes, disrupting of critical metabolic pathways and interaction with bacterial DNA.

The effects of silver, either as a metal (AgNPs) or in compounds is known to be non-specific, influencing many bacterial structures and metabolic processes at the same time (Figure 5). Among these are the following: inactivation of bacterial enzymes [202,203], disruption of bacterial metabolic processes [204,205,206] and the bacterial cell wall, accumulation in the cytoplasmic membrane and increase of its permeability [167,203,207], collapse the plasma membrane potential [206], interaction with DNA (deoxyribonucleic acid) [202] and generation of reactive oxygen species [208,209,210], which damage biomacromolecules [211]. Thanks to their multi-level mode of action, AgNPs destroy or inhibit the growth of pathogenic microorganisms including highly resistant bacterial strains at low concentrations from a few to several tens of mg/L [92,160,162,167,175,207]. Importantly, no relevant data describing bacterial resistance to AgNPs or inactivation of antibacterial action of AgNPs (nanoparticles) have been reported yet. Bacterial resistance to silver is driven only with the ionic form of silver and apart from others, was deeply researched by Silver *et al.* [212,213]. Bacterial resistance to ionic silver originated from clinical environments [214] and also from naturally occurring strains [215]. Besides reduction of Ag+ to a less toxic oxidation state, the probable Ag+ resistance mechanism involves an active efflux from the cell, by either P-type ATPases (adenosine triphosphatase) or chemiosmotic Ag+/H+ antiporters [216,217,218].

In recent years, a synergistic effect between AgNPs and various antibacterial agents has been investigated. Potara *et al.* studied antimicrobial activity of chitosan-coated AgNPs against two strains of *S. aureus* [219] and revealed that minimum inhibitory concentrations (MICs) of the composites were ten times lower than those of AgNPs and chitosan alone respectively. Another capping agent, myramistin increased activity of AgNPs against *E. coli* up to 20 times [220]. Combined treatments with a lactoferrin/xylitol hydrogel and silver-based wound dressings acted synergistically against the forming of biofilms of clinical wound isolates of methicillin-resistant *S. aureus* and *P. aeruginosa* [221]. Synergy of AgNPs and antimicrobial peptides polymyxin B and gramicidin S was reported also against different Gram-negative bacteria [222].

Recently several studies have indicated that AgNPs may strengthen the antibacterial effects of conventional antibiotics (beta-lactam antibiotics, macrolides, lincosamides, aminoglycosides) either additively or synergistically [223,224,225,226,227,228,229]. The synergistic effect of antibiotics and AgNPs was reported even at concentrations below their own effectiveness (*i.e.,* below MICs) [225,230,231,232,233]. Brown *et al.* showed a synergistic effect of AgNPs functionalized with ampicillin, even against multiple-antibiotic-resistant isolates of *P. aeruginosa, E. aerogenes* and methicillin-resistant *S. aureus* [234]. Also Smekalova *et al.* and Panacek *et al.* proved enhancement of the antibacterial effect of antibiotics in combination with AgNPs against several animal and human pathogens and resistant bacterial strains [235,236,237]. These findings clearly showed that it is possible to find an effective combination of antibiotics and AgNPs or another antimicrobial with a multi-level mode of action, resulting in a synergistic antimicrobial effect allowing efficient inhibition of bacterial pathogens including highly resistant bacterial strains using significantly lower doses as compared to an antibiotic alone. Replacement of frequently used antibiotics by AgNPs or a combination of these antibiotics with AgNPs represent a promising tool on how to kill bacteria without the development of antibiotic resistance [238].

## 10. Potential Side Effects of Silver Nanoparticles

The main problem by using AgNPs on biomaterials is that they are considered toxic not only for bacteria, but also to human host cells. Toxicity of silver nanoparticles to mammalian cells is considerably lower in comparison with antibacterial effective concentrations also due to the fact that eukaryotic cells have an antioxidant cellular mechanism that protects them [239,240]. The extended use of AgNPs can lead to a number of health problems from argyria [241] to silver accumulation in human liver and kidney. Although silver and its derivatives are already in clinical use, evidence of serious health problems [242] and high toxicity are rare [243].

A number of *in vitro* studies have been performed exploring the effects of AgNPs on a variety of cell types [88,239,244,245,246,247,248,249,250,251,252,253]. The most common mechanisms of toxicity from nanosized silver particles, as well as silver ions released from them [245,254] are: oxidative stress [246,255], Trojan-horse mechanisms [256,257] and DNA damage [163].

The question arises as to what determines if silver nanoparticles are toxic or not. In general, toxicity is determined by many factors, either on the side of the nanoparticles or on the side of the body that they are in contact with. Regarding nanoparticles, these are mainly size, shape [258], charge, surface modification, tendency to release ions, dose and exposure time. The role of the particle size is more important than concentration or dose [259,260] because smaller nanoparticles have a higher surface/volume ratio leading to higher oxidation and dissolution, accompanied by higher silver ion release [261]. Therefore, smaller AgNPs may show higher toxicity due to their larger specific surface area and associated faster Ag+ release compared to larger AgNPs [260]. However, that does not necessarily mean higher toxicity in a particular material and situation. Silver ion release is controlled by surface modification/stabilization and can be further influenced by other compounds presented in biological environment. Moreover, Ag+ release is also dependent on the formation of a protective oxidized silver layer that prevents full oxidation and dissolution of AgNPs [262]. Likewise, it is known that spherical nanoparticles are less toxic than wires [263] and negatively charged NPs exhibit low toxicity [264,265].

On the host side, the potential toxicity of AgNPs is determined by patient health status, routes of exposure, gender and other factors. In some organs (liver, kidney), silver is accumulated soon after application, while in others (brain, lung) higher concentrations are detected after a prolonged period [266,267,268,269]. Pauksch *et al.* [270] investigated the effect of AgNPs on human osteoblasts and it turned out that AgNPs were toxic at concentrations higher than 10 μg/g. The authors suggested that there is a gap between the toxic and antibacterial doses of AgNPs. This statement was confirmed by Necula *et al.* [271] who tested the antibacterial efficacy and toxicity towards the human osteoblastic cell line. They demonstrated that the antibacterial dose is by an order of magnitude lower than that having a toxic effect on human cells. These observations support the promising usage of presence of a therapeutically useful window for the application of AgNPs in orthopaedics.

## 11. Protocol for Testing of Silver Nanoparticle Coating Technologies Intended for Usage in Orthopaedics

There is no doubt that nanotreatment of biomaterial surfaces offers new opportunities for PJI prevention. On the other hand, the main obstacles preventing broader usage of such technologies are cytotoxicity and resultant decreased biocompatibility. It should be cautioned that nanotechnologies can also induce unintended inflammatory responses related to activation of immune cells such as dendritic cells, macrophages and others. Concern also exists over the mechanical properties of implant nanocoatings since damage may occur during surgical implantation, especially in cementless implants inserted via press-fit methods. In addition, creating a coating-substrate interface robust enough to sustain the mechanical stresses involved in surgical implant insertion and ultimate loading once *in vivo* remains a challenge. Lastly, the risk of remote effects of absorbed nanosilver is still a potential problem.

Therefore, a set of *in vitro* tests (followed by *in vivo* experiments) is required to characterize in detail antibacterial efficacy, as well as biocompatibility and safety of such material modifications. The latter means to examine the cytotoxicity, cancerogenicity, interactions with osteoblasts and other cells and the potential of adverse stimulation of an immune response. As a result, specialists in nano-toxicology (esp. nano-genotoxicology, cytotoxicity, immunotoxicity), *in vitro* pharmacokinetics, pharmacodynamics and kinetics of particles are needed to collaborate in the development, preclinical testing and approval of any material modifications for clinical usage.

### 11.1. Demonstration of Antibacterial Efficacy

A critical step in progress lies in the demonstrating that newly developed biomaterials, or surface modifications possess antibacterial efficacy [272]. To date there is no widely accepted methodology available that could precisely and reproducibly demonstrate antibacterial behaviour of the proposed anti-infective technologies. Major criticisms are levelled at the static “closed” testing system, whereas *in vivo,* the implant has to face a dynamic, continuously changing, mechanically unstable and predominantly fluid environment [273]. As a result, the majority of studies to date have used inappropriate and insufficient protocols.

Controllable, standardized testing conditions that closely mimic the human *in vivo* environment are needed in order to overcome the aforementioned issues [273]. PJIs develop under low shear conditions and a multidirectional low-pressure fluid flow. A variety of testing tools have been proposed that attempt to simulate conditions of continuous or intermittent fluid-displacement in both the low and high shear conditions [274]. Protocols for cultivation of particular species (multispecies) biofilms under controllable, constant and reproducible conditions have been also described [275]. Finally, representative *in vitro* and *in vivo* models to test bacterial adhesion and biofilm formation on biomaterials for each particular clinical situation (*i.e.*, total joint arthroplasty, internal, external fixation) should be further developed and appropriately validated. Given the large variability of antibacterial strategies, it is likely that testing methods must be better tailored to match the specific proposed strategy at hand [150].

### 11.2. Testing of Cytotoxicity

Although many studies presented new nanoparticle surface treatments proving *in vitro* safety [271], others demonstrated the potential danger of such materials [276]. Nanoparticles have different effects on human health depending on the bulk material from which they have been produced [277]. In addition to the elemental composition, factors like nanoparticle dose, size, shape, exposure time and surface chemistry can affect its biological behaviour. Regarding the shape, silver nanowires showed the strongest cytotoxicity and immunological responses, whereas spherical silver particles had negligible effects on cells when tested in human cells [278]. Liu *et al.* found that 5 nm AgNPs were more toxic than 20 and 50 nm AgNPs in four cell lines (A549, HepG2, MCF-7, SGC-7901), indicating a size-dependent effect on cell viability [253]. It should be noted that some cell lines (PC-12 and NIH-3T3) exhibit greater sensitivity to AgNPs than another mammalian cell lines [279]. The rate of ion release and its variation in different media should be taken in consideration as well. All this concludes that cytotoxicity testing should be always suited exactly for the proposed implant coating (*i.e.*, exact nanoparticle size, concentration, shape, fixation method *etc.*) and its intended use in a specific tissue. In addition, a level of cytotoxicity can be dependent on the assay technique and a difference between extraction-based and direct contact assays has been found [280].

When testing new materials or surface modifications, the cytotoxicity testing is performed first and other tests (anti-bacterial, immunoreactivity *etc.*) are advanced only after the biomaterial is classified as biologically not harmful. Cytotoxicity testing is rapid, sensitive and inexpensive. Another big advantage of cytotoxicity testing is the standardization of the procedure, ISO 10993 and the FDA (Food and Drug Administration) blue book memorandum (#G95-1) and its suitability for the testing of biomaterial from any part of the medical device (*i.e.*, TJA, internal, external fixation). This test is commonly performed using a mouse fibroblasts cell-line as target cells, following the exposure with the material (direct contact) as well as the extract of the material. Cells are very sensitive to biologically harmful extractables in certain quantities resulting in visible signs of toxicity, such as changes in cell morphology, vacuolization, or detachment. A different way of testing the nanotoxicity was described by Liu *et al.* via evaluation of induction of apoptosis [281].

Regarding novel anti-infective treatments with silver nanoparticles, a recent study reported that the BALB/c 3T3 cell line is 1000 times more sensitive for testing the toxicity of silver NPs than the *in vivo* animal models [282]. Although some studies showed a dose-dependent cytotoxic effect of nano-silver, new types of nano-silver were proven to be not cytotoxic [283], or it was shown that combination of a low amount of nano-silver with antibiotics provides an effective antibacterial action with negligible cytotoxic effect [236].

### 11.3. Testing of Immunoreactivity

Sensitization testing represents another part of the testing battery for new biomaterials, establishing the potential of a biomaterial to elicit immunogenic and allergenic responses (immunoreactivity). Currently, the most commonly used tests for novel materials and those medical devices that contact deep tissue, is the guinea pig maximization test (GPMT) where the extract of a biomaterial together with an adjuvant, is intradermally injected in model animals (Biological Evaluation of Medical Devices). Alternatively, a mouse local lymph node assay (LLNA) requiring less material than GPMT but needed to harvest the lymph nodes from sacrificed animals may be used with some precautions, such as a high number of false positives.

However, all animal tests are expensive and take days (weeks) to get results. It is therefore of great interest to replace or drastically reduce the utilization of tests based on experimental animals with suitable cell-based assays which exhibit required reliability, accuracy and importantly correlate to human reactivity. Several studies have shown great potential in the use of the MUTZ-3 human dendritic-cell cell-line for assessing *in vitro* sensitizing potency of chemicals and biomaterials, using a genomic biomarker signature [284,285,286]. Besides the MUTZ-3 assay, other tests are investigated for their potential to predict sensibilisation in humans as well [287]. However, it is likely that new testing methods must be validated and standardized to match the requirements for accuracy and ability, to be sensitive to the whole spectrum of molecules, with allergenic potential including nanosilver.

## 12. Time to Translation?

In the field of orthopaedics, there are no implants protected with silver nanotreatment available for clinical usage to date. At least two manufacturers already produce TJA treated by galvanic deposition of elementary silver on request (Implantcast GmbH–Medizintechnik, Buxtehude, Germany; Stanmore Implants, Borehamwood, UK). Initial clinical experiences with these “tailored” implants have been promising [288]. In addition, at least one study examined clinical usage of thermal-sprayed silver oxide in hydroxyapatite coating for total hip implants [84].

The situation is a little better in the field of indwelling medical devices protected by surface treatment with AgNPs. Again, in contrast to extensive experimental research, only several clinical studies have been conducted to demonstrate reduction of infections associated with the AgNP coating in these devices (left within a bodily organ for a limited time). For instance, the usage of external ventricular drainage catheters treated with AgNPs decreased the infection rate [289] while the venous catheters tailored with AgNPs failed to lower infection rates [290]. However, together these data preclude making any conclusions in support of their widespread clinical usage.

Examination of global grants and published studies of this topic suggests a striking discrepancy between proposed strategies of antibacterial surface treatment and ultimate completion of *in vitro* and *in vivo* experimentation. In fact, we believe that very little progress has actually been made in the translation of the aforementioned modalities into clinically useful technologies. Barriers to translational medicine in this area are not only related to economic, medicolegal and biotechnological issues but with major problems in the demonstration of the safety of clinical trials. Concerns about long-term durability of such new implants as compared to traditional implants are also realistic. Leaders in this field have recently proposed that in order for some of these obstacles to be overcome, we must improve efficiency and effectiveness amongst all the partners involved. Patients will benefit from these technologies only by improving collaborative efforts among governments, regulatory agencies, industry leaders and health care payers [291]. While pressures exist worldwide to diminish the incidence of PJIs, surprisingly there is not a single large clinical study examining the role of broad-range implementation of implants containing antibacterial surface treatments.

## 13. Future Developments

The ideal implant surface modification using whatever approach, should provide antibacterial protection throughout the life of the implant with minimal side effects. In relation to AgNPs and their usage in modification of implants, there are three crucial future developments. The first one is synthesis of AgNPs with defined optimal size, ensuring high antibacterial activity and concurrently low cytotoxicity to mammalian cells; that means good biocompatibility with tissues without acute or long-term adverse effects. The second is the development of a new coating technique, or improvement and optimization of a current one ensuring reliable formation of compact, continuous and durable layer of AgNPs. The third concerns the elimination of an inhibitory effect of human lipids and proteins preventing AgNPs from implementing their intended antibacterial effect. These substances cover surfaces of TJA immediately after an implant is placed into the human body. To meet this challenge, recent advances in the field of surface chemistry, fluid mechanics, fluid mechanobiology, bio-inspired materials and/or endogenous mechanisms of immune stimulation should be utilized. 

Another important issue related to antibacterial efficiency of AgNPs is connected with the possible development of bacterial resistance to silver NPs. It can be expected that with increasing use of AgNPs in killing bacteria or in the prevention of bacterial colonization in clinical medicine, the bacterial resistance to AgNPs could develop. As a result, strategies combining AgNPs with other antibacterial substances/approaches (either composite or nanocomposite layers), in order to achieve additive/synergistic effects are highly reasonable and should be investigated.

Finally, further investigation should be carried out in the field of strategies combining AgNPs with approaches restoring/maintaining local tissue homeostasis and modulating the immunologic surveillance and patrolling. This concept might comply with a wide variety of clinical situations ranging from residual low dose bacterial load during the surgery, to late haematogenic spreading of infection.

## 14. Conclusions

There is no doubt that prevention is the best response to the growing problem of orthopaedic implant infections. Engineers believe they are able to develop reliable, durable, non-toxic and safe biomaterials preventing bacterial adhesion and formation of biofilm on surfaces. Strategies incorporating nanopatterning and other nanotechnologies show great promise. Research in the field of antibacterial surface treatment has demonstrated *in vitro* and *in vivo* effectiveness of the technologies based on AgNPs, combining a strong antibacterial effect with relative inertness to the inner environment of a patient. On the other hand, issues relating to the mechanical properties of these technologies and the potential for detrimental side effects, such as toxicity and interference with osseointegration require further investigation.

## Figures and Tables

**Table 1:** Examples of anti-infective strategies proposed for treating of surfaces used in orthopaedic implants.

Strategy	Features	Examples	References
Prevention in adhesion and adsorption		Anti-adhesive polymers	[66,67,68]
		Albumin	[69]
		Super-hydrophobic surfaces	[70,71,72]
		Nano-patterned surface	[73,74,75,76,77]
		Hydrogels	[78,79,80,81]
		Silicon nitride ceramics	[82,83]
Methods to kill bacteria	Inorganic	Silver, silver-oxide	[84,85,86]
		Silver nanoparticles	[87,88,89,90,91,92,93,94]
		Gold nanoparticles	[95,96]
		Titanium dioxide	[97,98,99]
		Selenium ions	[100,101,102]
		Copper ions/nanoparticles	[103,104]
		Zinc ions	[105,106]
		Iodine coating	[107]
		Bioactive glass	[108,109]
		Graphene oxide	[110,111]
	Organic	Coated or covalently linked antibiotics	[112,113,114,115,116]
		Chitosan derivatives	[117,118,119,120]
		Signaling, inhibiting and antimicrobial peptides	[121,122,123]
		Cytokines	[124]
		Enzymes	[125,126]
	Other	Non-antibiotic bactericidal substances	[127]
	Combined	Multilayer coating	[128,129,130,131,132]
		Synergy material intensification	[133,134,135]
		Positively charged polymers	[136]
Multi-functional and smart coating	Passive	Nanostructured “smart” materials	[68,137,138,139,140]
	Active	Concept: sensors conjoined to nanocontainers	[141,142,143,144,145,146]
Alternative approach		Lytic bacteriophages	[147]
		Surface-adaptive anti-biofilm nanocarriers	[148]

## Abbreviations

The following abbreviations are used in this manuscript:

Ag	silver
AgNP	silver nanoparticle
ATPase	adenosine triphosphatase
DAL/CHI	dopamine-modified alginate/chitosan
DNA	deoxyribonucleic acid
EPS	extracellular polymeric substances
FDA	Food and Drug Administration
GPMT	guinea pig maximization test
LCC	local carriers or coating
LLNA	local lymph node assay
MAMP	microbe associated molecular pattern
MIC	minimum inhibitory concentration
MRSA	methicillin-resistant *Staphylococcus aureus*
MSCRAMM	Microbial Surface Components Recognizing Adhesive Matrix Molecule
NP	nanoparticle
NT	nanotube
PJI	prosthetic joint infection
PRR	pattern-recognition receptor
QAS	3-trimethoxysily-propyldimethyloctadecyl-ammonium chloride
QAS	quaternary ammonium salt
Ti	titanium
TiO_2_	titanium dioxide
TJA	total joint arthroplasty
USA	United States of America
UV	ultraviolet
